# Coverage, efficacy or dosing interval: which factor predominantly influences the impact of routine childhood vaccination for the prevention of varicella? A model-based study for Italy

**DOI:** 10.1186/s12889-016-3738-x

**Published:** 2016-10-21

**Authors:** Katsiaryna Holl, Christophe Sauboin, Emanuele Amodio, Paolo Bonanni, Giovanni Gabutti

**Affiliations:** 1 GSK Vaccines, Avenue Fleming 20, 1300 Wavre, Belgium; 2 University of Palermo, Via Del Vespro 133, 90127 Palermo, Italy; 3 University of Florence, Viale GB Morgagni, 48, 50134 Florence, Italy; 4 University of Ferrara, Via Fossato di Mortara 64b, 44121 Ferrara, Italy; 5 Present Address: Unit of Epidemiology, ATS Brianza, Viale Elvezia 2, 20900 Monza, Italy

**Keywords:** Varicella, Routine varicella vaccination impact, Coverage, Efficacy, Dosing interval

## Abstract

**Background:**

Varicella is a highly infectious disease with a significant public health and economic burden, which can be prevented with childhood routine varicella vaccination. Vaccination strategies differ by country. Some factors are known to play an important role (number of doses, coverage, dosing interval, efficacy and catch-up programmes), however, their relative impact on the reduction of varicella in the population remains unclear. This paper aims to help policy makers prioritise the critical factors to achieve the most successful vaccination programme with the available budget.

**Methods:**

Scenarios assessed the impact of different vaccination strategies on reduction of varicella disease in the population. A dynamic transmission model was used and adapted to fit Italian demographics and population mixing patterns. Inputs included coverage, number of doses, dosing intervals, first-dose efficacy and availability of catch-up programmes, based on strategies currently used or likely to be used in different countries. The time horizon was 30 years.

**Results:**

Both one- and two-dose routine varicella vaccination strategies prevented a comparable number of varicella cases with complications, but two-doses provided broader protection due to prevention of a higher number of milder varicella cases. A catch-up programme in susceptible adolescents aged 10–14 years old reduced varicella cases by 27–43 % in older children, which are often more severe than in younger children. Coverage, for all strategies, sustained at high levels achieved the largest reduction in varicella. In general, a 20 % increase in coverage resulted in a further 27–31 % reduction in varicella cases. When high coverage is reached, the impact of dosing interval and first-dose vaccine efficacy had a relatively lower impact on disease prevention in the population. Compared to the long (11 years) dosing interval, the short (5 months) and medium (5 years) interval schedules reduced varicella cases by a further 5-13 % and 2-5 %, respectively. Similarly, a 10 % increase in first-dose efficacy (from 65 to 75 % efficacy) prevented 2–5 % more varicella cases, suggesting it is the least influential factor when considering routine varicella vaccination.

**Conclusions:**

Vaccination strategies can be implemented differently in each country depending on their needs, infrastructure and healthcare budget. However, ensuring high coverage remains the critical success factor for significant prevention of varicella when introducing varicella vaccination in the national immunisation programme.

**Electronic supplementary material:**

The online version of this article (doi:10.1186/s12889-016-3738-x) contains supplementary material, which is available to authorized users.

## Background

Varicella is a highly infectious childhood disease caused by varicella-zoster virus (VZV). Each year, the total number of varicella cases in all age groups more or less equals the size of the birth cohort, in countries with no routine varicella vaccination (RVV) programme [1]. In addition, seroprevalence studies in Europe have shown that over 90 % of people have been infected with varicella by 10–15 years of age [2].

Many patients with varicella will visit a general practitioner (GP) and 2–6 % of those cases will have complications, while the annual incidence of varicella hospitalisations in Europe is 1.9 to 5.8 per 100,000 population [3]. The high infection rates and associated economic burden of varicella result in a significant public health burden [4].

Varicella infection can be prevented through effective varicella vaccines with well-established immunogenicity, reactogenicity and safety profiles [3]. In countries where RVV programmes have been implemented, a significant reduction in the incidence and burden of varicella has been observed [3]. Therefore, the World Health Organization (WHO) recommends that a RVV programme should be considered in countries where varicella has an important public health burden and where high (>80 %) coverage can be sustained [5].

With increasing constraints on healthcare budgets, policy makers need more clarity on which vaccination strategy to choose for optimal reduction of the disease burden. Several practical aspects that need consideration include how to reach and sustain high coverage levels in order to optimise protection against varicella disease. Other factors that influence the impact of vaccination include the choice of using either one-dose or two-dose vaccination schedules, either short (months) or long (years) intervals between two doses, and whether or not a catch-up programme should be implemented when introducing childhood RVV. RVV will be introduced in Italy as a national programme in the near future, following analysis of the vaccine data from eight pilot regions which have already implemented it. An Interregional Group on Varicella Vaccination (IGVV) was established in 2013 to assess the varicella vaccination effectiveness using common standardised methods. Since 2003, eight Italian regions (Basilicata, Calabria, Friuli - Venezia Giulia, Apulia, Sardinia, Sicily, Tuscany and Veneto) started to introduce childhood RVV (in children aged 13–15 months and 5–6 years) in their regional immunisation programmes, using different schedules. Currently all regions use a two-dose schedule [6].

The objective of this model-based study is to help policy makers choose an appropriate vaccination strategy in their country, by exploring the relative impact of several key factors on varicella burden: (1) coverage, (2) one-dose or two-dose schedule, (3) vaccine efficacy of the first dose, (4) short or long interval between doses, and (5) whether or not to implement a catch-up programme. Although this study aims to inform RVV policy makers in general, the example of Italy was used, given the diverse regional strategies currently in place and anticipated policy changes.

## Methods

An age-structured dynamic transmission model was developed, based on the framework described by Brisson et al. [7, 8] and following the same methodology used for the adaptation for France [9], to assess the impact of varicella vaccination on disease burden by varying the key influential vaccination strategy parameters. The model predicts both varicella and zoster diseases over a lifetime with and without vaccination to reflect the full picture of the disease burden related to VZV. The model structure shows varicella disease states as susceptible, latent or exposed, infectious, and recovered. Factors such as waning of vaccine efficacy over time and the influence of population migration on infection rates were included. The model was adapted to match the epidemiology of varicella prior to vaccination in Italy using an empirically-derived contact matrix [10]. A detailed description of the model can be found in Additional file 1. The incidence of varicella before vaccination was compared to the predicted incidence over time following the introduction of vaccination in the model. Based on these comparisons, the percentage reduction in varicella cases is presented post-vaccine introduction for all combinations of dosing, coverage and efficacy tested. After introducing childhood RVV, milder ‘breakthrough’ cases of varicella can occur in the model. The predicted incidence of varicella over time for each scenario is presented below.

### Model assumptions and scenarios (see Table 1)

**Table 1:** Scenario analyses

Parameter	Values	Source
Vaccination coverage: 1^st^ dose & 2^nd^ dose (VC D1_D2)	95–80 % 85–70 % 75–60 %	Assumptions
Vaccine efficacy (VE): for 1^st^ dose	65 % 75 %	Prymula et al. [11] Assumption
Vaccine efficacy (VE) for 2^nd^ dose	95 % (fixed)	Prymula et al. [11]
Dosing interval scenarios Age at 1^st^ dose & 2^nd^ dose	Short (1–1.5 years): 13 months & 1.5 years Medium (1–6 years): 13 months & 6 years Long (1–12 years): 13 months & 12 years	Assumptions
Catch-up programme	For susceptible 12-year-olds, for 11 years, 20 % coverage	Assumptions

The model assumed that the maximum vaccine coverage would gradually be attained in the first 3 years of introducing the RVV programme. Three vaccination coverage scenarios were considered for dose one & dose two; including high (i.e., 95 & 80 %), medium (i.e., 85 & 70 %) and low coverage (i.e., 75 & 60 %).

Two different values were assessed for vaccine efficacy of the first dose (i.e., 65 or 75 %). For two-dose schedules, vaccine efficacy following the second dose was fixed at 95 % [11].

Three scenarios were considered to assess the impact of a longer or shorter interval between the two vaccine doses; including a 5-month, a 5-year and an 11-year interval. These intervals were based on current Measles, Mumps and Rubella (MMR) schedules used in different countries, while in Italy several regions have typically used a 4-or 5-year interval between doses.

All dosing scenarios included a catch-up programme in adolescents (12-year-olds) who were still susceptible to varicella (i.e., not vaccinated and had not yet had varicella). The catch-up programme, with a coverage of 20 %, was modelled for the first 11 years since introducing vaccination. From the twelfth year, the original vaccinated cohort will have reached the age of 12 years, and would therefore be protected. A scenario presents the impact of not including a catch-up programme.

## Results

Increasing coverage or efficacy, or shortening the dosing interval had a positive effect on the prevention of varicella but each to a different extent. The impact of various vaccination strategies on varicella prevention are presented below.

### Routine varicella vaccination of toddlers and children: impact of the number of doses

**Fig. 1:**
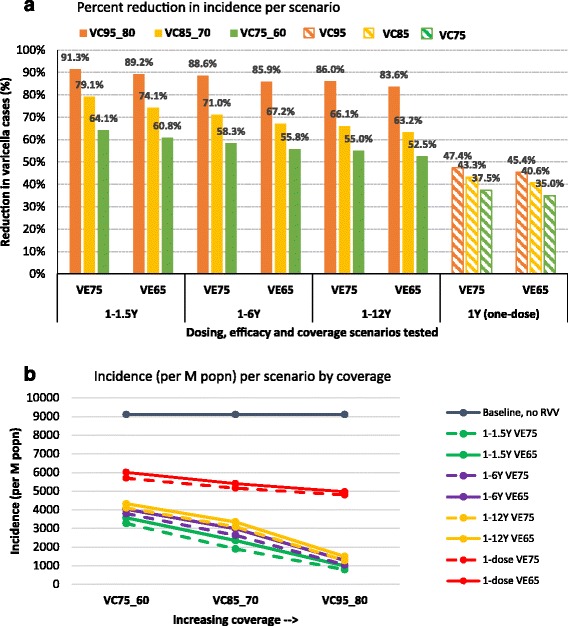
Percentage reduction in varicella incidence (**a**) and incidence per 100,000 popn (**b**) across all age groups at 30 years after vaccination, for different coverage, efficacy and dosing scenarios compared with no varicella vaccination. Legend: VC (vaccine coverage 1^st^ dose_2^nd^ dose), VE (1^st^ dose vaccine efficacy)

**Fig. 2:**
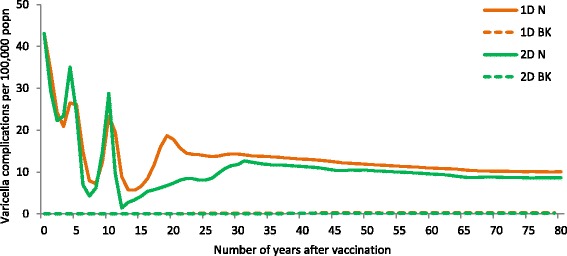
Complications among natural (N) and breakthrough (BK) varicella cases with a one-dose and two-dose vaccination strategy. Legend: 1D (one-dose), 2D (two-dose), N (natural), BK (breakthrough). Note: Complications among breakthrough cases were close to 0 with both one-and two-dose strategies

**Fig. 3:**
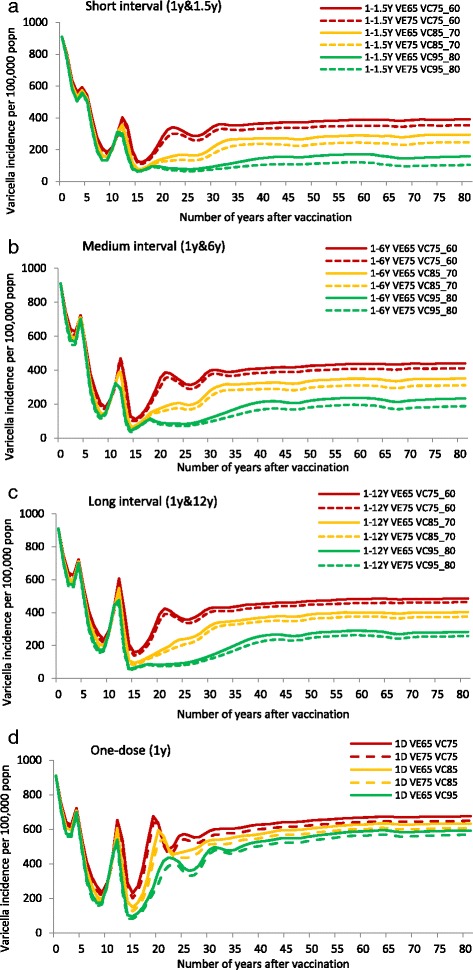
Varicella incidence (per 100,000 population) following RVV for each scenario. Legend: VE (vaccine efficacy), VC (vaccine coverage 1^st^ dose_2^nd^ dose), 1D (one-dose)

The first practical issue to consider when implementing RVV is the choice to use a one- or two-dose vaccination programme. As per our modelling results, a one-dose schedule effectively controls varicella disease albeit with the presence of mild breakthrough cases, whilst a two-dose schedule provides optimal protection against mild and severe varicella with the potential to reduce breakthrough varicella cases in the population. The baseline varicella incidence before introduction of RVV was 910.4 per 100,000 population per year. Varicella incidence decreased immediately after introduction of the vaccine, with by year 30 around 70 % reduction (incidence of 273.0/100,000) and around 40 % reduction (546.0/100,000) across all age groups for two- and one-dose strategies, respectively (Fig. 1). The overall number of complications due to varicella was 43 per 100,000 population per year. The reduction in number of complications in the first 15 years of post-introduction of vaccination was substantial (up to 90.1 % for two-dose and up to 84.6 % for one-dose strategies). Between 15 and 30 years post - introduction of vaccination, there was a peak in complications with one-dose strategies (up to 18.7/100,000 after 19 years), reflecting the peaks in incidence (Fig. 3), and due to accumulation of non-vaccinated varicella cases. However, this difference became minimal (6.0 %) between the two strategies after 30 years of vaccination (Fig. 2)

### Routine varicella vaccination of toddlers and children: impact of the coverage

Whether considering a one-dose or two-dose vaccination strategy, it is important to understand the impact of vaccination coverage on varicella disease burden in the population. Within each vaccination strategy considered in the model, it is apparent that high coverage was the key driver of vaccine impact on varicella (Fig. 1).

A low coverage reduced varicella by 64.1, 58.3 and 55.0 % (to 327, 380 and 410/100,000) with a two-dose strategy and short, medium and long dosing intervals, respectively. When coverage was high, an additional 27.2–31.1 % reduction in varicella cases was observed. The disproportionate improvement in outcomes (i.e., vaccinating 20 % more children provides around 30 % fewer varicella cases in the population) highlights the additional indirect benefit vaccination provides at higher coverage levels, by providing herd immunity (Figs. 1 and 3).

The effect of a 10 % increase from low to medium coverage and from medium to high coverage, respectively, was to reduce varicella cases by an additional 15.0 % (to 190.1/100,000) and 12.2 % (78.8/100,000) with the short dosing interval, 12.7 % (263.7/100,000) and 17.5 % (104.1/100,000) with the medium dosing interval, and, 11.1 % (309.1/100,000) and 19.9 % (127.4/100,000) with the long dosing interval (see Fig. 1).

### Routine varicella vaccination of toddlers and children: impact of the dosing interval

Within two-dose schedules, there is further debate about whether a short-term (5 months) or longer term (5 or 11 years) interval should be used.

Shortening the dosing interval resulted in an additional 3-8 % reduction in varicella cases (from 5 years dosing interval to 6 months) and 2-5 % reduction (from 11 years dosing interval to 5 years). The overall 5-13 % reduction in number of varicella cases was observed when 6 months between the two doses was considered instead of 11 years (Fig. 1). Changes in dosing interval appear to have a smaller impact on varicella burden than changes in coverage.

### Routine varicella vaccination of toddlers and children: impact of the first-dose efficacy

With a one-dose vaccination schedule and 75 % vaccine efficacy post-dose one, a 37.5 % (to 569/100,000) and 47.4 % (479/100,000) reduction in varicella cases was observed with low and high coverage, respectively. With 65 % efficacy post-dose one, the percentage of varicella cases prevented was 35.0 % (601/100,000) and 45.4 % (497/100,000), which is only slightly lower (2–3 %), showing that a 10 % drop in efficacy of dose one would not have a significant impact on overall disease prevention.

Similarly with a two-dose vaccination schedule and 75 % efficacy post-dose one, the reduction in varicella cases ranged from 55.0 % (low coverage and long interval) to 91.3 % (high coverage and short interval). This was comparable to the 52.5 % (low coverage and long interval) to 89.2 % (high coverage and short interval) reduction observed with 65 % efficacy post-dose one (Fig. 1).

### Routine varicella vaccination of toddlers and children: impact of a catch-up programme

**Fig. 4:**
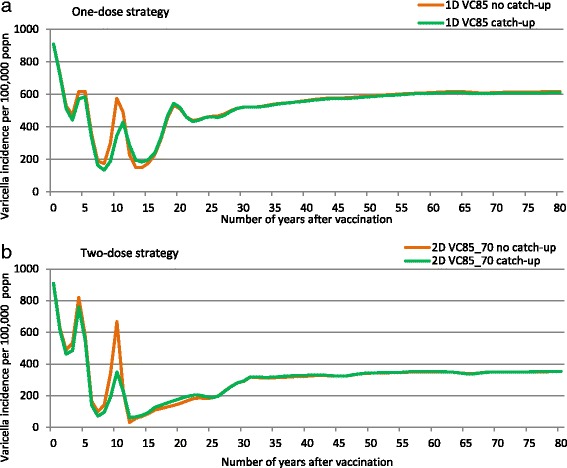
Influence of a catch-up programme on varicella incidence with a one-dose and two-dose strategy. Legend: 1D (one-dose), 2D (two-dose), VC (vaccine coverage 1^st^ dose_2^nd^ dose)

The impact of a catch-up programme on disease burden was assessed for both a one-dose and two-dose strategy, using a coverage of 85 % for the first dose and 70 % for the second dose. Figure 4 shows that the catch-up programme substantially reduced the second peak in incidence of varicella that is predicted to occur around ten years after introducing childhood RVV, by 47.4 % (to 350.8/100,000) with a two-dose strategy and by 25.5 % (to 428.4/100,000) with a one-dose strategy. Without introduction of catch - up programme the incidence of varicella at around 10 years post - introduction of vaccination was 667.6 and 575.2/100,000 for a two-dose and one-dose strategy, respectively (Fig. 4a and b).

**Fig. 5:**
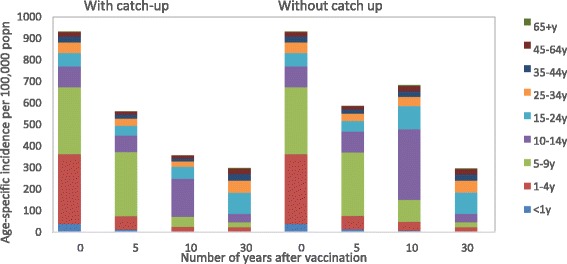
Influence of a catch-up programme on age-specific incidence of varicella (two-dose scenario). Legend: y (year). Note: The vaccine strategy in Fig. 5 included dosing at 1y and 6y with coverage of 85 and 75 % respectively

Figure 5 shows the breakdown of varicella cases by age group predicted to occur at given time points after introducing childhood RVV, with and without a catch-up programme, using the example of the two-dose strategy. The impact of the catch-up programme ten years after introducing childhood RVV was to significantly reduce the overall incidence of varicella (by 47.4 %, from 667.6 to 350.8/100,000), as well as to reduce the incidence in the 10–14 year age group (by 79.9 %, from 94.8 to 19.1/100,000) compared with having no catch-up programme.

## Discussion

The WHO position is that countries should consider introducing varicella vaccination into the routine immunisation programme where varicella is an important public health burden. They recommend that these countries have sufficient resources to maintain high coverage. Currently, eight countries recommend childhood RVV in the European Union, either at the national or regional level, while sixteen countries recommend targeted vaccination of susceptible teenagers or at-risk groups [3]. Several practical factors related to vaccination strategy need to be taken into consideration as they play a key role in determining the impact of a RVV programme on varicella cases in the population; such as how to optimise coverage, choice of dosing strategy and whether to implement a catch-up programme.

Following the introduction of childhood RVV, there is a period when incidence rates fluctuate, this is common with infectious disease models which typically assume homogenous contacts and transmission risk within each age group and include a dynamic relation between the force of infection, the proportion of infectious and susceptible individuals. In our case, other factors can influence these variations such as the highly infectious nature of varicella, the changes in coverage rates over time and the temporary implementation of a catch-up programme.

This study considered many possible vaccination scenarios such as one- or two-dose schedule, different coverage rates, dosing intervals, efficacy post-dose one and availability of a catch-up programme to predict the reduction of varicella burden in the population following a childhood RVV programme. The results of this analysis have shown that the most influential factor in reducing varicella incidence was high coverage. A shorter interval between two doses improved outcomes further, while the impact of a 10 % change in first-dose efficacy was less significant whether with a one- or two-dose strategy.

Varicella vaccination programmes have been implemented in different ways, with some countries implementing a childhood RVV programme while other countries target only high-risk groups. Among countries with a RVV programme, a one-dose schedule has been publicly funded in Australia since 2005 [12] with a coverage of over 80 % resulting in important reductions in varicella morbidity and mortality [13, 14]. Whereas in the USA and Germany, the initial one-dose schedule was replaced by a two-dose schedule after some years, resulting in even greater disease prevention in the population [15, 16]. Breakthrough varicella is thought to be caused mainly by primary vaccine failure rather than vaccine efficacy waning [17]. This could explain why countries implementing a two-dose schedule were able to further reduce the incidence of breakthrough cases compared to those using one-dose schedules [3]. Data from active surveillance in the USA show that a one-dose schedule was able to significantly decrease the number, size and duration of varicella outbreaks, however the addition of a second dose was able to nearly eliminate outbreaks [3]. When comparing a one-dose and two-dose strategy in this study, both were found to significantly reduce the burden of varicella, however the impact with a two-dose strategy was greater. Although a one-dose vaccination strategy prevented fewer varicella cases, its prevention of severe cases (i.e., with complications) was comparable to two-dose strategies. Evidence from countries with childhood RVV supports the finding that one-dose strategies have higher efficacy against more severe than less severe varicella, while two-dose strategies have high efficacy against any varicella regardless of severity [3]. Therefore, the main benefit of a one-dose schedule appears to be in reducing mortality and severe morbidity, while a two-dose schedule has been found to reduce the disease burden irrespective of severity and to prevent cases of breakthrough infection [18].

Maintaining high vaccine coverage is critical to providing substantial protection for the population, whether with a one-dose or two-dose vaccination strategy, given the highly infectious nature of VZV. The model findings are consistent with WHO recommendations in that sustaining coverage of 85 % or higher with a RVV programme will have an optimal impact. Our findings show that a high coverage was also able to compensate for other vaccination strategies which may be less effective at reducing varicella burden, such as longer interval between doses and lower vaccine efficacy post-dose one. In countries where childhood RVV has been implemented with high coverage rates, surveillance data have shown significant and rapid reductions in varicella disease burden (cases, complications, hospitalisations and deaths) observed in all age groups including non-vaccinated age groups such as infants and adults, demonstrating herd protection effects [3].

This study found that shortening the dosing interval increased the number of cases prevented, although the effect was less important than increasing coverage rate. Another benefit of a short interval between doses is that this could help reduce the risk of breakthrough varicella in the interval between doses [17]. In Italian regions where childhood RVV has already been introduced, the first dose is given around the 13^th^ to 15^th^ month of life and the second dose around the 5^th^ to 6^th^ year of life; thus with a 4- to 5-year interval between doses [6]. There is evidence from three recent Italian studies showing that a long-interval schedule does not significantly affect the performance of a two-dose varicella vaccination programme [6, 19, 20]. These studies reported very high rates of disease reduction across eight regions of Italy (Apulia, Basilicata, Calabria, Friuli-Venezia Giulia, Sardinia, Sicily, Tuscany and Veneto) that implemented childhood RVV between 2003 and 2013; overall incidence decreased from 6.7 per 1000 population in 2003 to 1.7 per 1000 population in 2012. In Sicily, which was the first Italian region to implement varicella vaccination, there was a 95 % reduction in cases over 10 years, with coverage rates increasing to 85 % [20]. So while a shorter interval favours fewer cases of varicella, the largest driver of prevention is higher coverage. In the USA, despite a long interval between doses [21], a clear benefit of vaccination was observed with a high coverage of 90 % for infants targeted for vaccination [22, 23]. This supports the finding that coverage is a more important factor than dosing interval in determining the success of a childhood RVV programme. When deciding on dosing schedules, countries should therefore consider fitting in varicella vaccinations with their current immunisation schedule, by assessing the number of vaccines currently given at different ages and the timing of visits, to help improve coverage for their RVV programme. Combining the second dose of varicella vaccination with a scheduled visit for other childhood vaccinations could help to increase coverage of the second dose.

The choice of using MMR with a single antigen varicella vaccine (MMR + V) or the combined MMRV depends on country-specific preferences, given the uncertainty surrounding potential increased febrile convulsion risks with the first dose of MMRV. An association was seen in clinical trials and observational studies between the administration of the first dose of MMRV and febrile convulsions, compared with MMR + V vaccination [24, 25]. German data found one additional case of febrile convulsion with MMRV per 5882 or 2747 vaccinees compared with MMR or MMR + V, respectively [24, 26]. Other studies also found a small increased risk of febrile convulsions in the second week post-vaccination [26–28]. By contrast, the region of Tuscany in Italy decided to keep MMRV for the first dose as they had not observed an increased risk of any adverse events due to MMRV compared with MMR + V [29]. In another study, the incidence febrile convulsions in the 40-day post-vaccination period did not differ between MMR + V and MMRV. There was a minimal impact of MMRV on the overall population risk of febrile convulsions, with an excess risk of 3.52 febrile convulsions per 10,000 vaccinees compared to MMR + V [27]. There was no change in the baseline incidence of febrile convulsions in the population as a whole following the introduction of MMRV. The small increased risk of febrile convulsions occurring within the first 2 weeks of administration of MMRV vaccine must be balanced against administering separate MMR and varicella vaccines [26, 28], potentially impacting vaccine uptake [30]. The eight Italian regions that provided RVV offered two doses of MMRV, MMR + V or single antigen V, given at different times and sometimes in combination with appointments for different vaccines [6]. Coverage is linked to dosing strategy; in countries where there is a high MMR coverage, switching to MMRV does not appear to affect coverage, but switching to MMR + V tends to result in a lower V coverage. In Germany, the uptake of varicella vaccination (but not MMR) decreased by 4–19 % when the national recommendations changed from the administration of MMRV to MMR + V [30]. Benefit/risk assessment suggests that the use of MMRV instead of MMR + V can prevent an additional 1976 varicella-related hospitalisation days per year at the cost of an additional 225 vaccine-related febrile convulsions hospitalisation days when coverage drops by 12 %. That is the trade-off between the two vaccination schemes that needs to be considered when making decisions on their use in immunisation programmes [31].

The primary goal of a varicella vaccination programme is to generate a public health impact by reducing the number of varicella cases and related hospitalisations and deaths. In 2000, using mathematical models, Brisson et al. [7] showed a temporal immediate increase in herpes zoster (HZ) incidence post-vaccination. However, medium-term epidemiological evidence is now available from several countries that have implemented varicella vaccination. The majority of these studies concluded that HZ incidence was either not increasing, or the increase was not directly related to varicella vaccination [13, 32, 33], or that the study was not designed to prove causality between HZ and varicella vaccination [34, 35]. Factors that may have influenced an increase in HZ incidence include increasing elderly population [36], increased oral corticosteroid use [37], and, chronic comorbid conditions [38, 39]. Therefore this paper focuses solely on the impact of influential factors on varicella.

Limitations of this study relate to the underlying model assumptions for which a lack of evidence exists; such as duration of immunity after two doses or waning of natural immunity and two-dose vaccine efficacy for full or partial or non-responders. In order to focus on the influential factors of interest for this study, the same simplified catch-up programme was implemented in all dosing scenarios. Although a conservative catch-up coverage of 20 % was used, the catch-up duration was 11 years based on the needs for the long-interval dosing schedule (i.e., after 11 years the first infants vaccinated would receive their second *routine* dose), and may therefore have artificially improved the outcomes in this dosing scenario. Despite this, the long-interval dosing scenarios had the worst predicted outcomes (among two-dose schedules). Therefore, the effect of using a simplified catch-up programme on results was expected to be minor in long-interval dosing scenarios. In this study, despite the low coverage of the catch-up programme, it was found to be beneficial in reducing the peak of cases predicted to occur around ten years after implementing RVV among older children in both one-dose and two-dose strategies. This finding has also been observed in other modelling studies of RVV [3]. All Italian regions may introduce a catch-up programme for susceptible adolescents, since this was offered by the National Vaccination Plan in 2012–2014, although coverage of the catch-up programme may vary across the different regions [6, 20].

The impact of different vaccination scenarios on payers’ budget and cost-effectiveness ratios have not been assessed in the current study. Scenarios with higher coverage, more doses and catch - up will have increased vaccination programme cost, however, this increase needs to be balanced against expected larger cost savings due to better disease prevention (e.g., reductions in resource utilisation and lost productivity). Published economic evaluations have found that introducing both one- and two-dose RVV strategies are cost-effective from payer perspectives and even cost-saving from societal perspectives [40].

## Conclusion

The current study may provide insights for policy makers on how to optimally reduce the burden of varicella in their country. Coverage of varicella vaccination is a critical factor in significantly reducing the number of varicella cases, whether for a one-dose strategy or a two-dose strategy. The combination of high coverage with dosing intervals of five years or less was more effective at preventing varicella than longer dosing intervals. A high coverage can compensate to some extent for decreases in cases prevented due to long intervals between doses. Similarly, at a lower coverage, varicella prevention was improved with short interval dosing schedules. Coverage had a greater impact on vaccination outcomes than the efficacy of the first dose or the dosing interval. The catch-up programme substantially reduced the peak in cases after ten years of introducing childhood RVV, even with a modest vaccine coverage in adolescents.

## Additional file

Additional file 1: Technical appendix - dynamic transmission model for Italy. (DOCX 299 kb)

## Abbreviations

1D: One-dose
2D: Two-dose
BK: Breakthrough cases
GP: General practitioner
HZ: Herpes zoster
MMR: Measles, mumps and rubella
N: Natural cases
RVV: Routine varicella vaccination
USA: United States of America
VC: Vaccine coverage of dose 1_dose 2
VE: Vaccine efficacy
VZV: Varicella-zoster virus
WHO: World Health Organization
y: Year
