# Supplementary material for: Coverage, efficacy or dosing interval: which factor predominantly influences the impact of routine childhood vaccination for the prevention of varicella? A model-based study for Italy
Source: BMC Public Health. 2016 Oct 21;16:1103. doi: 10.1186/s12889-016-3738-x (PMC5073465; doi:10.1186/s12889-016-3738-x)
Supplement: Additional file 1: — Technical appendix - dynamic transmission model for Italy. (DOCX 299 kb) [file 12889_2016_3738_MOESM1_ESM.docx]

**Additional file 1 – Technical appendix - dynamic transmission model for Italy**

**Model description**

An age-structured dynamic transmission model developed in Matlab software (version R2013A) using the same basic structure as the models by Brisson *et al.*[^[[1]](#endnote-1)^,^[[2]](#endnote-2)^] was adapted and fitted to the seroprevalence of varicella in Italy without vaccination using an empirical contact matrix. The model predicts both varicella and zoster diseases over a lifetime with and without vaccination to reflect the full picture of the disease burden related to varicella-zoster virus (VZV), however, outcomes of the model on herpes zoster are not discussed here. All details of a similar dynamic model adapted for France are reported in Ouwens et *al.*[^[[3]](#endnote-3)^].

The model structure shows varicella disease states as susceptible, latent or exposed, infectious, and recovered; and zoster disease states as susceptible, infectious, and recovered. The normal disease progression (before vaccination) is shown in black, and the post vaccination disease states are shown in red (*Additional file - Figure 1*). After vaccination, people can become fully-protected or partially-protected and primary vaccine failures are shown to remain in the varicella susceptible state. After two doses of varicella vaccination, most partially-protected vaccine recipients become fully-protected. Breakthrough cases are assumed to be milder than naturally-acquired cases and to result in lower infection and reactivation rates.

**Model inputs**

The key model inputs used for the Italian adaptation are given in *Additional file - Table 1.* All input parameters and vaccine assumptions used in the model are given in  *Additional file - Table 2*.

Briefly, birth rate was calculated from Italian national statistics as a fraction of the annual birth cohort out of the total Italian population. The force of infection (FOI) was estimated based on a pooled estimate of age-specific Italian incidence data divided into 8 age groups (ages 1 year, 1–4 years, 5–9 years, 10–14 years, 15–24 years, 25–44 years, 45–64 years, and 65 years). It was assumed that on average 98% of individuals 65 years of age and older will have had varicella and that the proportion of susceptible individuals in each age group decreases exponentially. The *Who Acquires Infection From Whom* (WAIFW) matrix was based on the empirically-derived contact matrix from Mossong *et al*. [^[[4]](#endnote-4)^] for Italy. The rate of VZV reactivation was age-dependent and estimated to match the Italian zoster incidence data. The proportion of primary vaccine failures (corresponding to subjects with no seroconversion) was assumed to be 5% [^[[5]](#endnote-5)^]. Vaccine-induced immunity was assumed to wane over time, with an average duration of 17 years after 1 dose [^[[6]](#endnote-6)^] and lifelong protection after 2 doses.

**Calibration and model fit to Italian data**

In order to validate the WAIFW matrix, this matrix was used to estimate the FOI. The estimated FOI was found to be comparable to the FOI derived from the pooled data, using the resulting ‘percent susceptible by age’. A second validation of the matrix was performed: the results (percent susceptible) derived from running the dynamic model with the WAIFW matrix, were compared to the pooled published estimates. The results show that the WAIFW matrix provides a very close estimation of the published data (*Appendix Table 3*).

**Appendix Table 1 Key model inputs to adapt the model for Italy**

| **Parameter** | **Source** |
| --- | --- |
| Demographic parameters | Istat [^[[7]](#endnote-7)^] |
| Varicella seroprevalence | Gabutti *et al.* 2008 [^[[8]](#endnote-8)^] |
| Contact matrix | Mossong *et al.* 2008 [5] |
| Force of infection^‡^ | Bonanni *et al.* 2008 [^[[9]](#endnote-9)^], Coudeville *et al.* 2004 [^[[10]](#endnote-10)^], Nardone *et al.* 2007 [^[[11]](#endnote-11)^], Thiry *et al.* 2004 [^[[12]](#endnote-12)^], Gabutti *et al.* 2008 [8], Giaquinto *et al*. [^[[13]](#endnote-13)^], Salmaso *et al*. [^[[14]](#endnote-14)^] |

^‡^A pooled estimate was used to derive the force of infection for Italy. It was assumed that these data have a lognormal distribution.

**Appendix Table 2 Model parameters**

| **Parameter** | **Description** | **Value** | **Source** |
| --- | --- | --- | --- |
| **Demographic parameters** | | | |
| Birth | Fraction of annual birth cohort/total Italian population | 0.00937 | ISTAT [7] |
| **Biological parameters** | | | |
| σ | Latent period of varicella (avg 14 days) | 26.07 | Brisson *et al.* 2000 [1] |
| α | Infectious period of varicella (avg 7 days) | 52.14 | Brisson *et al.* 2000 [1] |
| δ | Waning natural immunity (avg 10 years) | 0.1 | Expert opinion |
| g * λ (a) | Exogenous boosting against zoster (100%) | 100% * λ | Brisson *et al.* 2000 [1] |
| **Vaccine parameters** | | | |
| Introduction time | Number of years before maximum vaccination coverage is reached | 3 | Assumption |
| Tv (1^st^ dose) | Varicella vaccine efficacy (% successfully vaccinated and temporarily protected) | 65% or 75% | 65% (Prymula *et al.* 2014 [5]) |
| P (1^st^ dose) | Varicella vaccine failures (%) | 5% | Prymula *et al.* 2014 [5] |
| 1-Tv-P (1^st^ dose) | Varicella vaccine-recipients partially protected (%) | 20% or 30% | 100%-Tv-P |
| 2^nd^ dose efficacy | Varicella vaccine efficacy (dose 2) for protected | 100% | Assumption |
|  | Varicella vaccine efficacy (dose 2) for partial responder | 89% | Assumption |
|  | Varicella vaccine efficacy (dose 2) for primary failure | 75% | Assumption |
| Wv1 | Waning rate for 1 dose of varicella vaccine (duration 17 years) | 0.0588 | Silverman *et al.* 2009 [6] |
| Wv2 | Waning rate for 2 doses of varicella vaccine (lifelong protection) | 1e^-6^ | Expert opinion |
| Ki * λ (a) | Rate of exogenous boosting | 0.91 * λ (a) | Brisson *et al.* 2000 [1] |
| h | Relative VZV reactivation after varicella vaccination | 0.167 | Brisson *et al.* 2000 [1] |
| b * λ (a) | Rate of infection among vaccinated susceptibles | 0.73 * λ (a) | Brisson *et al.* 2000 [1] |
| m | Relative infectiousness of infected vaccine-recipients versus non-vaccine-recipients | 0.5 | Brisson *et al.* 2000 [1] |

Note: VZV, varicella zoster virus

**Appendix Table 3 Estimates of percent susceptible**

| Age group | Overall estimate | Estimated ML* | Model estimate |
| --- | --- | --- | --- |
| 0 | 95% | 95% | 96% |
| 5 | 59% | 60% | 60% |
| 10 | 28% | 28% | 28% |
| 15 | 19% | 19% | 18% |
| 25 | 12% | 11% | 11% |
| 45 | 3% | 3% | 3% |
| 65 | 1% | 1% | 1% |
| 80 | 1% | 1% | 1% |

*ML: Maximum likelihood to feed the model parameters

**Additional file - Figure 1 Model Structure (Reproduced with permission [3]**


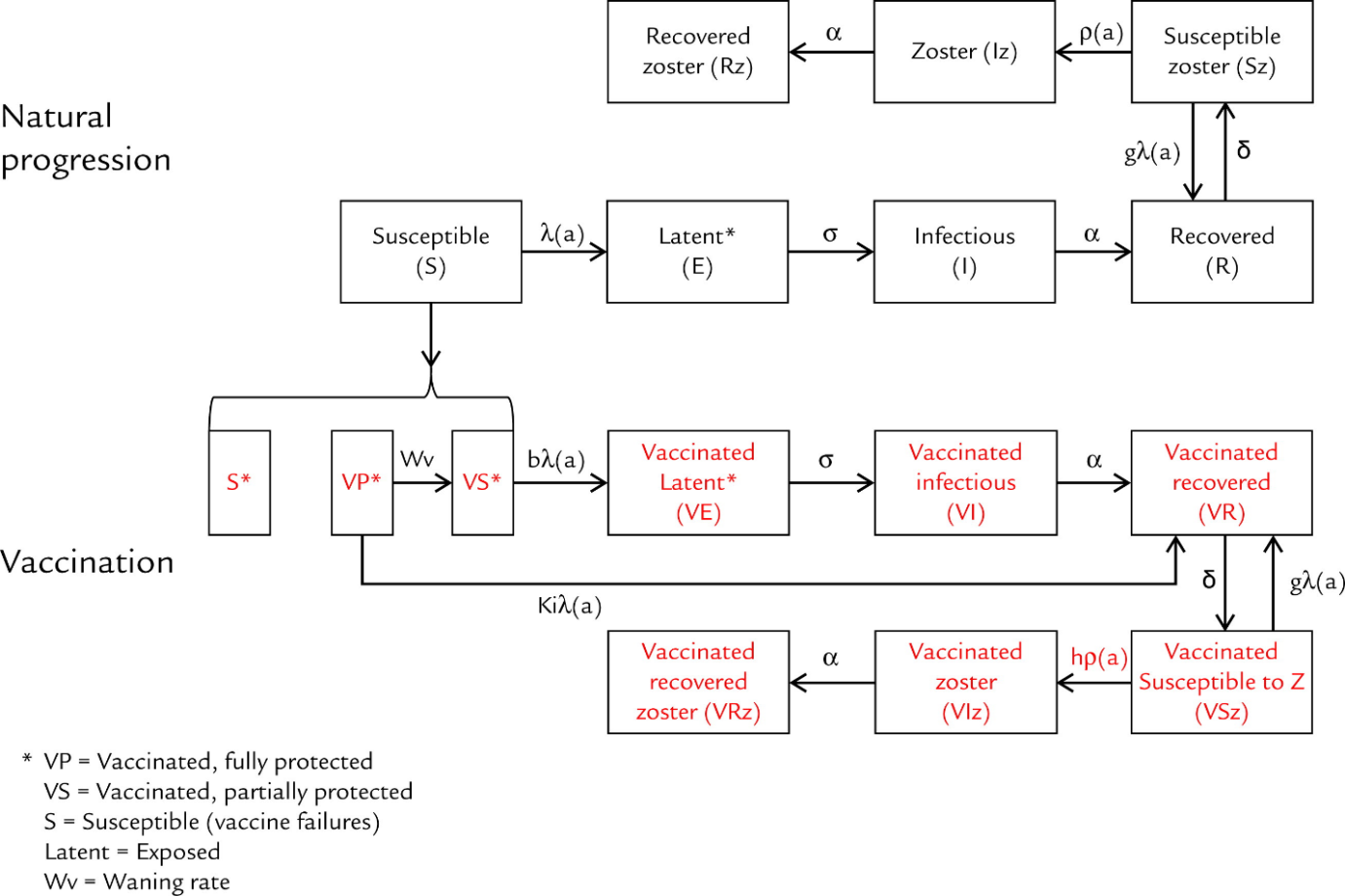


Legend: VP (Vaccinated, fully protected), VS (Vaccinated, partially protected), S (Susceptible, vaccine failures), Latent = Exposed, Wv = waning rate

Note: all other letters are described in Additional file – Table 2

**List of abbreviations**

**FOI:** Force of infection, **ML:** Maximum likelihood, **M popn:** Million population, **S:** Susceptible, vaccine failures, **VP:** Vaccinated, fully protected, **VS:** Vaccinated, partially protected, **VZV**: varicella-zoster virus, **WAIFW**: Who Acquires Infection From Whom, **Wv:** waning rate, **y:** year

# **References**

1. Brisson M, Edmunds WJ, Gay NJ Law B, De Serres G. Modelling the impact of immunization on the epidemiology of varicella zoster virus. Epidemiol Infect 2000; 125:651-69. [↑](#endnote-ref-1)
2. Brisson M, Melkonyan G, Drolet M, De Serres G, Thibeault R, De Wals P. Modeling the impact of one- and two-dose varicella vaccination on the epidemiology of varicella and zoster. Vaccine. 2010 Apr 26; 28(19):3385-97. [↑](#endnote-ref-2)
3. Ouwens MJ, Littlewood KJ, Sauboin C, Téhard B, Denis F, Boëlle PY, Alain S. The impact of 2-dose routine measles, mumps, rubella, and varicella vaccination in france on the epidemiology of varicella and zoster using a dynamic model with an empirical contact matrix. Clin Ther. 2015 Apr 1;37(4):816-829 [↑](#endnote-ref-3)
4. Mossong J, Hens N, Jit M, Beutels P, Auranen K, Mikolajczyk R *et al*. Social Contacts and Mixing Patterns Relevant to the Spread of Infectious Diseases. Plos Medicine, March 2008; vol 5, Issue 3; e74. [↑](#endnote-ref-4)
5. Prymula R, Bergsaker MR, Esposito S, Gothefors L, Man S, Snegova N *et al*. Protection against varicella with two doses of combined measles-mumps-rubella-varicella vaccine versus one dose of monovalent varicella vaccine: a multicentre, observer-blind, randomised, controlled trial. Lancet. 2014; 383:1313–1324. [↑](#endnote-ref-5)
6. Silverman B, Hemo B, Friedman N. Varicella vaccine effectiveness in an Israeli health maintenance organisation. Abstract presented at: 27^th^ Annual Meeting of the European Society for Paediatric Infectious Diseases; June 9–13, 2009; Brussels, Belgium. [↑](#endnote-ref-6)
7. ISTAT 2008: Italy’s National Census Bureau. Available at: http://www.indexmundi.com/g/g.aspx?c=it&v=29. Accessed on 22-Sep-2014. [↑](#endnote-ref-7)
8. Gabutti G, Rota M, Guido M, De Donno A, Bella A, Ciofi degli Atti ML *et al*. and the Seroepidemiology Group. The epidemiology of Varicella Zoster Virus infection in Italy. BMC Public Health 2008, **8**:372 [↑](#endnote-ref-8)
9. Bonanni P, Boccalini S, Bechini A, Banz K. Economic evaluation of varicella vaccination in Italian children and adolescents according to different intervention strategies: the burden of uncomplicated hospitalised cases. Vaccine. 2008 Oct 16; 26(44):5619-26. [↑](#endnote-ref-9)
10. Coudeville L, Brunot A, Giaquinto C, Lucioni C, Dervaux B. Varicella Vaccination in Italy. An Economic Evaluation of Different Scenarios. Pharmacoeconomics 2004; 22 (13): 839-855 [↑](#endnote-ref-10)
11. Nardone A, de Ory F, Carton M,  Cohen D, van Damme P, Davidkin I *et al*. The comparative sero-epidemiology of varicella zoster virus in 11 countries in the European region. Vaccine 25 (2007) 7866–7872 [↑](#endnote-ref-11)
12. Thiry N, Beutels P, Tancredi F, Romanò L, Zanetti A, Bonanni P *et al*. An economic evaluation of varicella vaccination in Italian adolescents. Vaccine 2004: 22(27-28): 3546–62 [↑](#endnote-ref-12)
13. Giaquinto C, Sturkenboom M, Mannino S, Arpinelli F, Nicolosi A, Cantarutti L; Gruppo di studio sulla varicella in età pediatrica (Pedianet Varicella Study Group). [Epidemiology and outcomes of varicella in Italy: results of a prospective study of children (0-14 years old) followed up by pediatricians (Pedianet study)]. Ann Ig. 2002; 14(4 Suppl 6):21-7 [↑](#endnote-ref-13)
14. Salmaso S, Mandolini D, Scalia Tomba G, Esposito N. La prevenzione della varicella in Italia: strategie di vaccinazione [Prevention of varicella in Italy: vaccination strategies].  Ann Ig. 2002; 14 (4 Suppl 6): 35-44 [↑](#endnote-ref-14)
